# Whipstitch and Locking Stitch Show Equivalent Elongation and Load to Failure Across 3 Suture Systems in a Biomechanical Model of Quadriceps Tendon Grafts for Anterior Cruciate Ligament Reconstruction

**DOI:** 10.1016/j.asmr.2024.100968

**Published:** 2024-07-01

**Authors:** Miguel A. Diaz, Eric A. Branch, Jacob G. Dunn, Anthony Brothers, Steve E. Jordan

**Affiliations:** a Foundation for Orthopaedic Research & Education, Tampa, Florida; b Tallahassee Orthopedic Clinic, Panama City, Florida, U.S.A.; c St. Francis Orthopaedic Institute, Columbus, Georgia, U.S.A.; d Andrews Institute for Orthopaedics & Sports Medicine, Gulf Breeze, Florida

## Abstract

**Purpose:**

To compare the biomechanical properties of quadriceps tendon (QT) graft stitch methods using 3 different suture systems for anterior cruciate ligament reconstruction.

**Methods:**

A total of 48 QTs were harvested from cadaveric knee specimens (age: 73 ± 7 years; range, 66-86 years). Samples were randomly divided into 3 groups where different suture needle systems were used to create 2 stitch methods: whipstitch (WS) and locking stitch (LS). Surgeons performed each technique to 5 stitches, each 0.5 cm apart. Stitching time was recorded. Samples were preconditioned and then underwent cyclic loading, followed by load to failure. Stiffness (N/mm), ultimate failure load (N), peak-to-peak displacement (mm), elongation (mm), and failure displacement (mm) were recorded.

**Results:**

WS and LS were equivalent across stiffness, ultimate load, and peak-to-peak displacement within groups 2 and 3. In group 1, the LS was stiffer than the WS, but the WS achieved a higher ultimate load. For all groups, the LS achieved lower elongation and failure displacement than the WS, with significant differences in groups 1 and 2. Within each stitching method, equivalence was determined for total elongation and ultimate failure load for all 3 suture system groups. For WS samples, group 1 all failed from suture breakage, and both groups 2 and 3 had instances of failure from suture pull-through. All LS samples failed from suture breakage.

**Conclusions:**

Both LS and WS provide adequate mechanical properties in each of the 3 suture systems. Differences in performance do exist; however, each method shows equivalent total elongation and ultimate failure load for all 3 suture systems. LS may be preferred over WS due to lower mean elongation and failure displacement.

**Clinical Relevance:**

There is an increased use of QT grafts in for anterior cruciate ligament reconstruction. However, there have been a limited number of studies comparing various stitching methods and optimizing techniques for QT graft fixation. This study may provide important information to surgeons about which suture techniques have better biomechanical profiles.

The quadriceps tendon (QT) autograft is commonly used for anterior cruciate ligament reconstruction (ACLR) because of its robust size and versatility as a graft.^1^ An abundant cross-sectional area makes doubling or quadrupling the graft unnecessary, and the QT’s average length consistently surpasses the 70 mm that is required for the all-inside technique.^1^, ^2^, ^3^, ^4^ As such, predictability of size on preoperative imaging has been cited as a benefit of the QT along with decreased graft site morbidity and improved versatility over more common autografts, including bone–patellar tendon–bone (BPTB) and hamstring tendon (HT) autografts.^5^

The QT graft has not been studied as extensively as the BPTB, HT, and semitendinosus tendon (ST) autografts for ACLR, but studies have cited the QT’s increasing popularity, rising from 2.5% in 2010 to 11% in 2014.^6^^,^^7^ If this rate is projected, assuming a linear growth rate, one can extrapolate to 2024 and estimate a use of 32%. An editorial published in 2024 commented that use of QT grafts has grown 30%.^8^ A systematic review of 20 studies concluded that there was no difference between full-thickness and partial-thickness QT autograft and that both were efficacious in primary ACLR.^5^ Results after revision ACLR are commonly known to be less favorable than those after primary ACLR.^9^^,^^10^ Eggeling et al.^9^ evaluated a small population of patients who underwent revision ACLR and concluded that their QT technique significantly lowered failure rates, improved Tegner and International Knee Documentation Committee scores, and reduced visual analog scale for pain score compared with the HT graft technique.

Clinically, the performance of a graft construct can be evaluated by surgeons based on patient outcomes, efficiency (time to prepare), or intraoperative preloading. In a benchtop setting, performance of suture constructs can be described through biomechanical characteristics such as ultimate load, elongation, stiffness, and failure mode.^11^

With the rise in the use of QT grafts, several biomechanical studies have emerged evaluating the mechanical properties and performance against various graft types and stitching methods. Hart et al.^12^ compared the biomechanical properties of BPTB, HT, and QT grafts and concluded that there was no significant difference in the ultimate load to failure and that the QT graft had greater stiffness compared to HT. Urchek and Karas^13^ compared the QT graft to a 6-strand HT and concluded similar biomechanical properties with respect to ultimate load to failure. While comparison between graft tissue types is imperative, the other key variable to consider is the security of the graft-suture interface, which is an important component for fixation.^14^ Suturing techniques require use of a needle to repeatedly pass suture through the tendon, but stitching can often be complex and time-consuming.^15^ For example, stitch methods that require multiple needle passes typically take more time and create more needle holes, increasing the risk of damage to the tissue.^16^ Moreover, the suture site presents as a stress riser, which has been cited in various biomechanical studies as the cause of failure and inconsistent repairs/reconstruction.^7^^,^^15^^,^^17^^,^^18^

The most common stitch methods currently used to secure quadriceps tendons in modern ACLRs include the whipstitch (WS) and the locking stitch (LS).^19^ Of locking stitch methods, the Krackow stitch (KS) has long been considered the gold standard.^14^ However, several new method adaptations proposed have investigated locking loop stitch, nonlocking loop stitch, or needleless methods.^15^^,^^18^, ^19^, ^20^, ^21^ Diaz et al.^11^ investigated the performance of different stitching methods (WS and whip-lock [WL]) across 2 tendon graft types (ST and QT) utilizing a novel suturing device that minimizes the number of needle passes. The WL is a hybrid stitch technique enabled by a 2-part needle. It creates a locking mechanism in the sutures, like the Krackow, but 1 pass of the needle enables suturing of both sides of the tissue, like the whipstitch. They concluded that the WL provided superior biomechanical properties in the ST graft, increasing the ultimate load to failure and stiffness compared to the WS. Interestingly, it was shown that either stitch method provided sufficient biomechanical performance in the QT, which may reflect the robust nature of the QT. These studies have shown that different techniques influence the maximum load to failure and elongation.^22^, ^23^, ^24^ Yet, there are a limited number of investigations comparing various stitch methods and optimizing techniques for the QT. A greater understanding of the techniques for ACLR with QT would assist clinicians in optimizing their approach and improving patient outcomes.

The purpose of this study was to compare the biomechanical properties of QT graft stitch methods using 3 different suture systems for ACLR. The biomechanical properties of each construct were compared for all 3 groups. It was hypothesized that whipstitch and locking stitch graft constructs will achieve equivalent biomechanical performance across the systems, and the novel system will reduce stitching time.

## Methods

### Graft Harvest and Specimen Preparation

A total of 48 QTs (n = 48) with a standard length of 7 cm were harvested from 48 cadaveric knee specimens (age: 73 ± 7 years; range, 66-86 years), which were stored at –20°C and thawed at room temperature for 24 hours before dissection, instrumentation, and testing. Tissue dissection, graft harvesting, and biomechanical testing was performed at the Foundation for Orthopaedic Research and Education. Specimen instrumentation was completed at the Andrews Research and Education Foundation. Tendons were cleaned and visually evaluated for the presence of tears or other abnormalities. The tendons were randomly divided into groups according to Table 1.

**Table 1:** Testing Groups

Group	Sample Size	Method	Representative image
1 Winter Innovations	8	WS EasyWhip	
	8	LS EasyWhip	
2 Arthrex	8	WS FiberLoop	
	8	LS FiberWire	
3 CONMED	8	WS SutureLoop Hi-Fi Suture	
	8	LS Hi-Fi Suture	
Total	48		

LS, locking stitch; WS, whipstitch.

Stitching was performed by 2 fellowship-trained orthopaedic surgeons (A.B. and S.E.J.). Surgeons used products from the 3 different systems to create whipstitch and locking stitch patterns. Cadaveric tendon samples were placed on a graft preparation stand. A skin marker was used to identify stitch placement along the center of the tendon. Five stitches were placed on one end approximately 0.5 cm apart. Stitching time for the 5-stitch series was recorded for all samples.

Length, width, and thickness for all tendons were measured with a digital caliper, where on average the grafts were 7.0 ± 0.7 cm, 12.7 ± 4.2 mm, and 8.5 ± 1.7 mm for length, width, and thickness, respectively.

### Materials and Group Design

**Fig 1:**
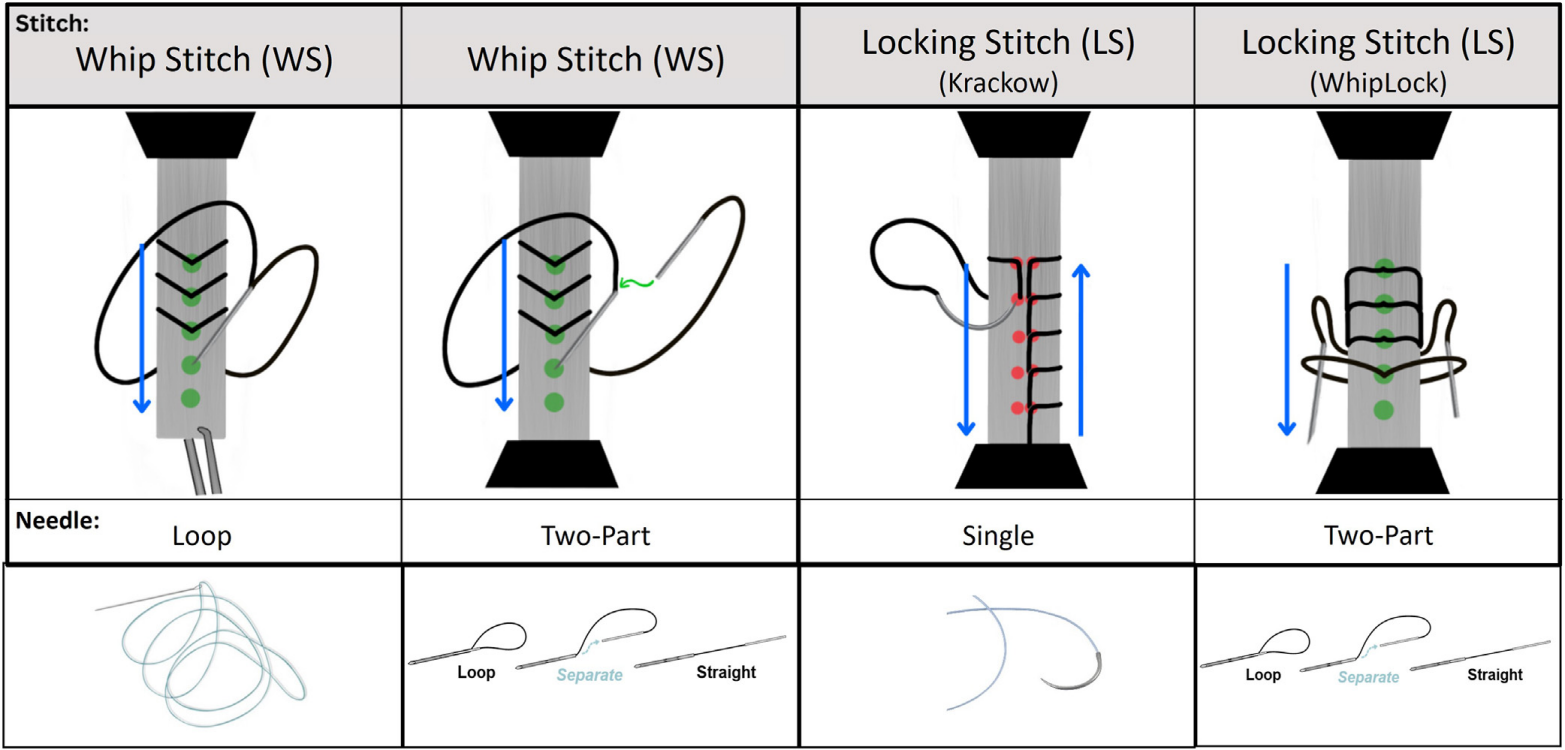
Illustration of different stitching methods and examples of suture types utilized.

Three different systems (groups 1-3, outlined below) were each used to create 2 stitch methods (whipstitch and locking stitch), as shown in Figure 1.

Group 1 (Winter Innovations) consists of a No. 2 EasyWhip used to create both a WS and a LS (specifically, WL); group 2 (Arthrex) consists of a No. 2 FiberLoop used to create a WS and a No. 2 FiberWire used to create a LS (specifically, KS); and group 3 (CONMED) consists of a No. 2 SutureLoop Hi-Fi used to create a WS and a No. 2 Hi-Fi used to create a LS (specifically, KS).

Products from systems in group 2 and group 3 are conventional needles that have been commonly used in the industry. A loop needle is typically used to create a whipstitch, and a single needle is typically used to create a locking stitch.

The suture system from group 1, EasyWhip, is a 2-part needle that consists of an insert that slides in the back end of a needle tip. When the tip and insert portions are connected, it creates a loop of suture. When they are separated, the suture is straight.^10^ This system can be used to create both a whipstitch or a locking stitch, whereas other systems require different products to complete the different methods. The WL is a cross between a whipstitch and a Krackow stitch. It creates a locking suture mechanism (like a Krackow) but requires half as many needle holes through the tissue (like a whipstitch).

### Biomechanical Testing

Biomechanical evaluation was established using previously published^15^^,^^20^^,^^21^^,^^25^, ^26^, ^27^, ^28^, ^29^ testing protocols. Cyclical testing was performed using a servohydraulic testing machine (MTS Bionix; MTS) equipped with a 5-kN load cell. The tendon graft was coupled to the MTS actuator by passing it through a cryoclamp cooled by dry ice to a temperature of –5°C (monitored by temperature probe).

**Fig 2:**
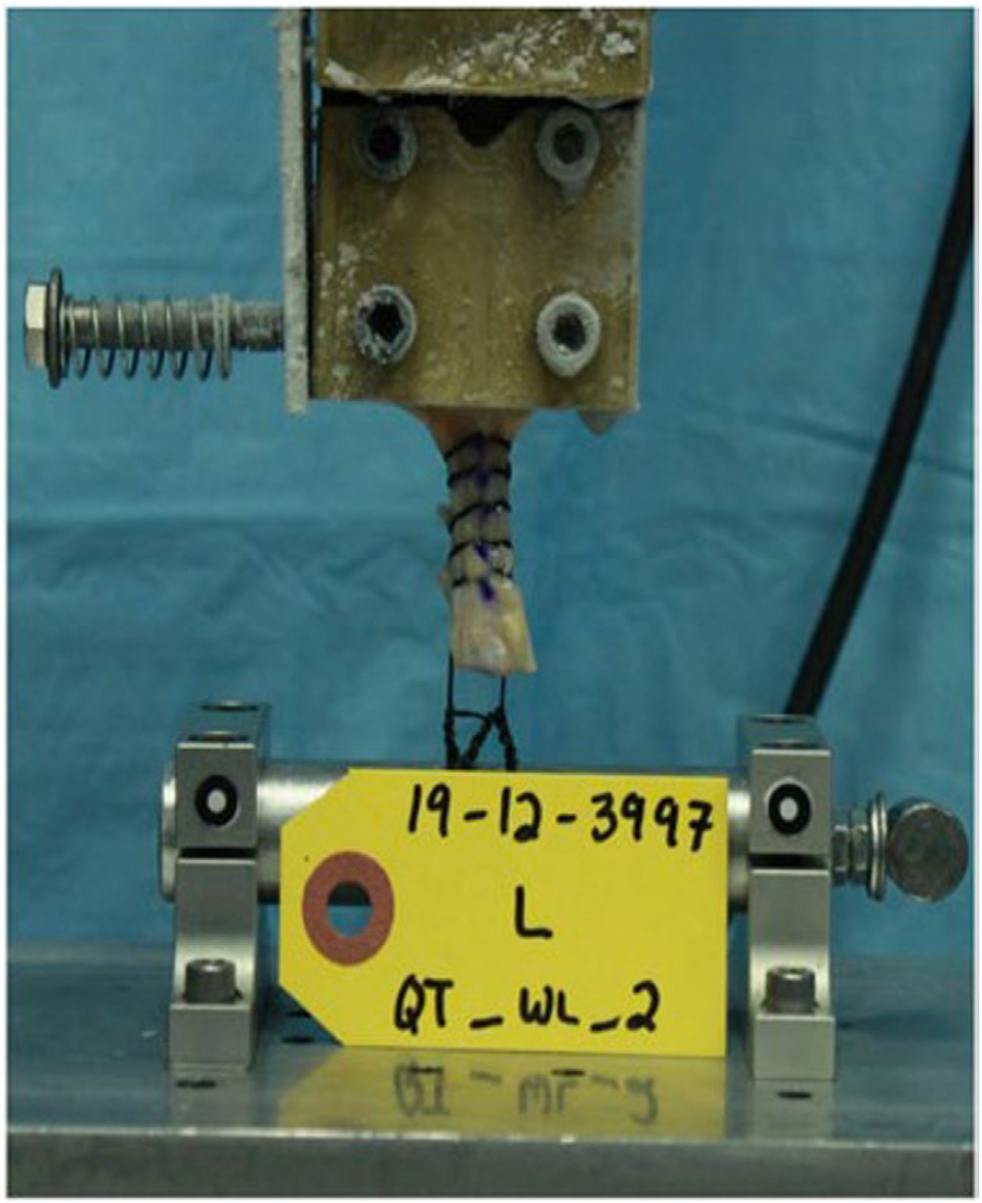
Biomechanical testing setup. The sample is under load in this image.

The 2 free ends of the suture were secured around the cylinder, which was rigidly fixed to the base of MTS, with 6-throw square knots.^28^^,^^29^ Length of suture loop, tendon grip length, and length of frozen tendon were standardized and measured across all specimens, where the cryoclamp was placed 1 cm above the first stitch, the total length of tendon exposed was 4 cm, and the length of suture to the cylinder was 2 cm (Fig 2). Before testing, a visual check was performed, along with the use of a temperature probe to verify the tendon within the cryoclamp was frozen. All testing samples were then preconditioned to normalize viscoelastic effects and testing variability through application of cyclical loading to 25 to 100 N for 3 cycles. The samples were then held at 50 N for 1 minute. Thereafter, the samples were loaded to 50 to 200 N for 500 cycles at 1 Hz.^15^ During cyclic loading, displacement data were collected from the actuator’s Linear Variable Differential Transformer at cycles 1, 10, and 50 and every 100 cycles as a measure of progressive construct elongation (mm). If samples survived cyclical loading, ramp-to-failure testing at 20 mm/min was performed.

During ramp to failure, stiffness (N/mm), ultimate failure load (N), ultimate failure displacement (mm), and failure mode were recorded. Total elongation was defined as the difference in y-displacement between the first cyclic peak and the last cyclic peak, whereas peak-to-peak displacement was defined as the average of the maximum and minimum displacement across the last 3 cycles. Stiffness was defined as the linear portion (slope) of the load-displacement curve, and failure was defined as the first significant decrease in the monotonically increasing force profile. Specimens were visually monitored for any slipping within the clamp during testing as well as on posttest analysis of the load-displacement curve to ensure that slipping of tendon within the clamp did not occur. Ultimate failure load was defined as the peak load at the onset of failure, and ultimate failure displacement was the corresponding displacement at the point of failure. Failure mode was defined as tissue pull-through or suture breakage.

Using mean and variance data based on displacement and failure load from prior studies^15^^,^^25^^,^^28^^,^^29^ of similar scope, a large effect size for each metric (effect size *d* of 1.4) was used for an a priori power analysis. Using a nonparametric design, with a significance threshold of .05, powers the study at the .83 level with a total sample size of 48 (8 samples per group) (G∗Power V3.1.9.2; Franz Faul).

### Data Analysis

A Wilcoxon rank-sum test was performed to identify differences in biomechanical properties (peak-to-peak displacement, total elongation, stiffness, ultimate failure load, and failure displacement) within each tendon graft type across the 2 stitch constructs (WS and LS). Moreover, a Kruskal-Wallis test was performed with post hoc analysis using the Steel-Dwass method to compare the biomechanical properties of each stitch construct and stitching time. Data are presented as mean ± standard deviation. All statistical comparisons were performed with JMP (JMP Pro 16, 2021; SAS Institute) at a significance level of α = 0.05.

## Results

### Donor Demographics

No statistical differences for age, height, or weight between groups were found.

After harvesting of the QT, all samples were measured, and no differences were found between the length, width, and thickness of tendons. For all right-sided tendons, the average length was 7.3 ± 0.4 cm, width was 12 ± 3.3 mm, and thickness was 8.8 ± 1.6 mm. Similarly, the left-sided tendons were 7.1 ± 0.6 cm in length, width was 12 ± 4 mm, and thickness was 8.7 ± 1.5 mm. Refer to Table 2 for additional details regarding outcomes.

**Table 2:** Data Summary for Stiffness, Load to Failure, Peak-to-Peak Displacement, Elongation, and Failure Displacement

Study Group	Construct	Mean Stiffness, N/mm	Mean Peak Load, N	Mean Peak-to-Peak Displacement, mm	Mean Elongation, mm	Failure Displacement, mm
Group 1	Locking stitch	75.2 ± 11.2	343.2 ± 22.3	2.1 ± 0.9	25.8 ± 9.5	45.7 ± 10.8

	Whipstitch	63.5 ± 8.4	378.9 ± 31.2	2.1 ± 0.2	35.6 ± 9.8	64.8 ± 12.9
	*P* value	.015	.018	.932	.069	.020

Group 2	Locking stitch	103.7 ± 23.4	369.1 ± 30.3	1.0 ± 0.1	14.2 ± 2.3	33.0 ± 3.4

	Whipstitch	103.1 ± 11.2	412.3 ± 102.6	1.6 ± 1.2	32.3 ± 18.3	66.2 ± 16.6
	*P* value	.318	.227	.245	.029	.001

Group 3	Locking stitch	79.3 ± 10.3	419.3 ± 31.5	1.4 ± 0.1	28.7 ± 5.1	60.2 ± 11.3

	Whipstitch	80.1 ± 32.4	437.6 ± 62.5	1.4 ± 0.3	32.8 ± 7.8	72.7 ± 10.5
	*P* value	.985	.558	≥.999	.280	.094

### Group 1

The average peak-to-peak displacements between the whip-lock locking stitch (LS_1_) and whipstitch (WS_1_) methods were not significantly different (*P* = .932). The WS_1_ constructs had more elongation when compared to the LS_1_, but this difference was not found to be statistically significant (*P* = .069). The stiffness of LS_1_ was significantly greater than WS_1_ (*P* = .015). Interestingly, the ultimate failure load for the WS_1_ was significantly larger than the LS_1_ (*P* = .018). The displacement at failure for WS_1_ was significantly larger than LS_1_ (*P* = .02).

### Group 2

For group 2, the average peak-to-peak displacements between the Krackow locking stitch (LS_2_) and whipstitch (WS_2_) were also not significantly different (*P* = .245). The WS_2_ constructs had significantly more elongation when compared to the LS_2_ (*P* = .029). No differences were found between the stiffness of LS_2_ and WS_2_ (*P* = .318). Similarly, no differences were found between the ultimate failure loads for the WS_2_ and the LS_2_ (*P* = .227). The displacement at failure for WS_2_ was significantly larger than LS_2_ (*P* = .001).

### Group 3 (G3)

The average peak-to-peak displacements between the Krackow locking stitch (LS_3_) and whipstitch (WS_3_) were not significantly different (*P* ≥ .999). The WS_3_ constructs had more elongation when compared to the LS_3_ but was found to not be statistically significant (*P* = .280). No differences were found between the stiffness of LS_3_ and WS_3_ (*P* = .984). Similarly, no differences were found between the ultimate failure loads for the WS_3_ and the LS_3_ (*P* = .558). No differences were found between the displacement at failure for WS_3_ and LS_3_ (*P* = .094).

### Group 1 Versus Group 2 Versus Group 3

When comparing across the different types of suture configurations, some equivalences and differences did arise between the 3 groups.

For the WS method, the peak-to-peak displacement was significantly higher in group 1 when compared to group 2 (*P* = .0173), but no differences were detected between group 1 and group 3. The total elongation was found to be equivalent across all groups (group 1: 36 ± 10 mm; group 2: 32 ± 18 mm; group 3: 33 ± 8 mm). The stiffness of group 2 (103 ± 11 N/mm) was significantly larger than group 1 (64 ± 8 N/mm; *P* = .001), whereas stiffness of WS by group 1 was equivalent to group 3 (80 ± 32 N/mm). The ultimate failure load was equivalent across all WS groups (group 1: 379 ± 31 N; group 2: 412 ± 103 N; group 3: 438 ± 63 N).

For the LS method, the peak-to-peak displacement was also significantly higher in group 1 when compared to group 2 (*P* = .014), but no differences were detected between group 1 and group 3. The total elongation (group 1: 26 ± 10 mm; group 2: 14 ± 2 mm; group 3: 29 ± 5 mm), stiffness (group 1: 75 ± 11 N/mm; group 2: 104 ± 23 N/mm; group 3: 79 ± 10 N/mm), and ultimate load (group 1: 343 ± 22 N; group 2: 369 ± 30 N; group 3: 438 ± 63 N) were found to be equivalent across all groups.

### Failure Mode

**Fig 3:**
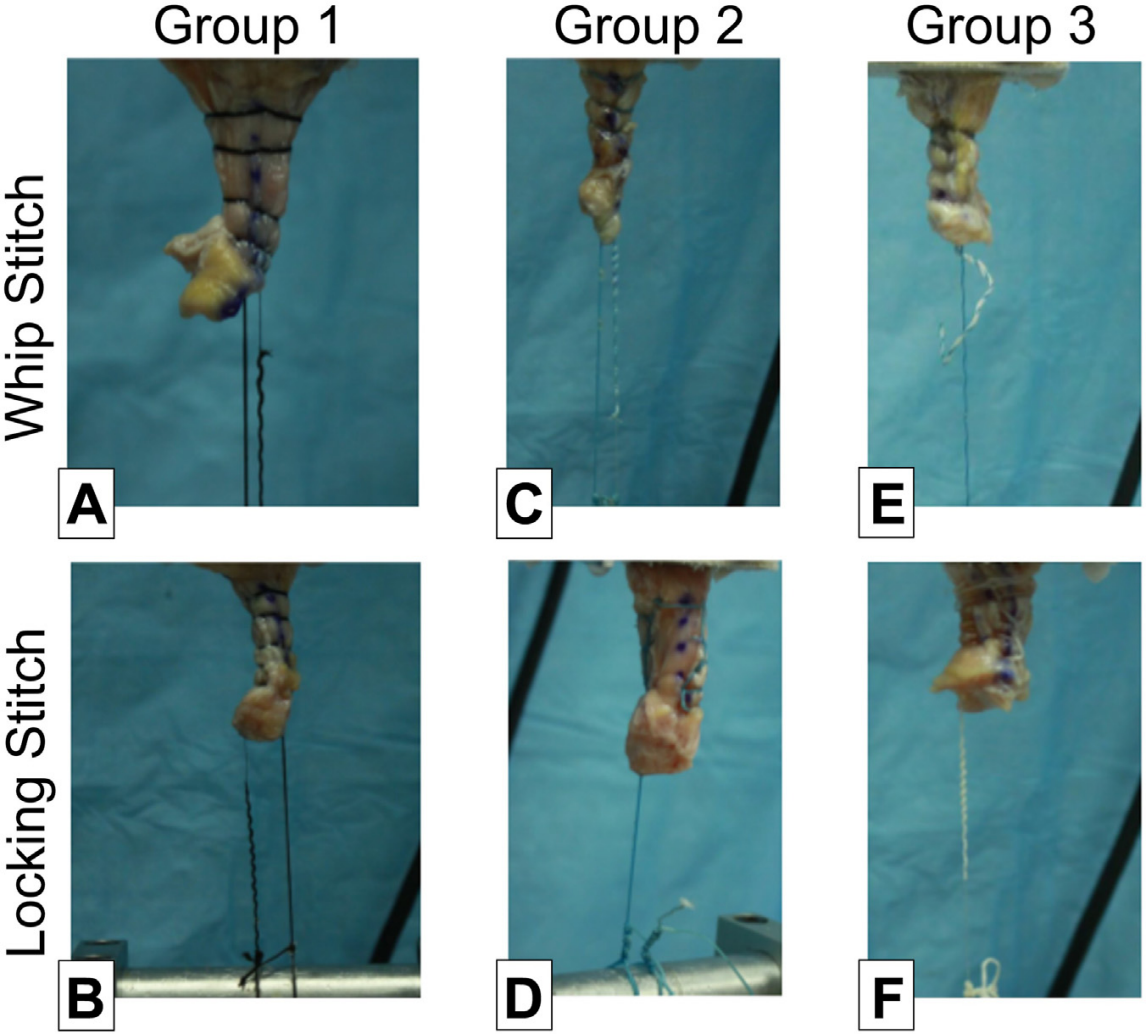
Representative failure modes for group 1, whipstitch (A), whip-lock (B); group 2, whipstitch (C), Krackow (D); and group 3, whipstitch (E), Krackow (F). Tissue strangulation and suture breakage were observed where the whipstitch groups had more strangulation as suture pulled through the tendon.

Common failure mode was suture breakage, followed by tendon tear or combination (Fig 3). The suture configurations (WS and LS) in group 1 all failed through suture breakage. Similarly, the LS configuration for both group 2 and group 3 all failed through suture breakage. For the WS configuration, group 2 had 1 instance that failed by suture pull-through and 1 instance of combined tendon tear and suture breakage. When looking at group 3, the WS configuration had 2 instances where the failure had a combined tendon tear and suture breakage. The failure mode for all groups can be seen in Table 3.

**Table 3:** Failure Modes by Test Group

		Failure Modes, %		
Study Group	Construct	Suture Pull-Through	Suture Breaking	Tendon Tear, Then Suture Breaking
Group 1	Locking stitch	—	100	—
	Whipstitch	—	100	—
Group 2	Locking stitch	—	100	—
	Whipstitch	12.5	75	12.5
Group 3	Locking stitch	—	100	—
	Whipstitch	—	75	25

### Timing

When comparing the time to perform the WS across the 3 groups, it was found that group 1 had a significant time savings (56 seconds faster) compared to group 2 (*P* = .0457). No significant differences were found between group 1 and group 3, or group 3 and group 2. For the LS, group 1 took significantly longer than group 3 (*P* = .015). No significant differences were found between group 1 and group 2, or group 3 and group 2 (Table 4).

**Table 4:** Grafting Time for Each Stitch Construct

Study Group	Total Time (min:s)
Group 1 (WS)	1:52
Group 2 (WS)	2:48
Group 3 (WS)	2:35
Group 1 (WL)	3:10
Group 2 (WL)	2:30
Group 3 (WL)	1:59

WL, whip-lock; WS, whipstitch.

## Discussion

In this cadaveric quadriceps tendon model, the main findings were that differences in biomechanical performance exist between the 3 suture systems; however, equivalence within each stitching method across all suture systems was determined for 2 critical metrics: total elongation and ultimate failure load. For both groups 2 and 3, the WS method had instances of failure from suture pull-through, whereas the LS methods failed from suture breakage. In contrast, both the WS and LS samples in group 1 all failed by suture breakage, and no instances of suture pull-through were observed. This may have clinical implications as it is important to minimize damage to the tendon and preferred that during excessive loads experienced by repair, the suture material will yield rather than the tendon. For the WS, it was determined that group 1 was significantly faster than group 2, by roughly 1 minute. Group 1 was also faster than group 3, but the time difference was not statistically significant. However, the 43-second time savings may be clinically significant. For the LS, it was determined that the speed of group 1 was equivalent to group 2 but significantly slower than group 3. These outcomes partially support our hypothesis that the whipstitch and locking stitch graft constructs wil achieve equivalent biomechanical performance across the systems, and the novel system will reduce stitching time and suggest group 1 to be a viable alternative.

The strength and performance of graft suturing configurations can be influenced by the suture materials, quality of the tendon, and stitch method. Group 2 utilized the Arthrex suture (No. 2 FiberWire), which is constructed from an ultra-high molecular weight polyethylene (UHMWPE) core with a braided jacket of polyester and UHMWPE. This differs from the material used to construct the sutures in group 1 and group 3, Winter Innovations (No. 2 EasyWhip) and CONMED (No. 2 Hi-Fi), respectively, which are both constructed from coreless braided 100% UHMWPE. The other factor that can account for performance differences are the tendon graft properties, but this may play a minimal role given no differences were detected for donor demographics, graft quality, or size. The distinction in suture materials may explain the variances for the peak-to-peak displacements and stiffness in the WS groups. When assessing the LS methods, stiffness was found to be equivalent despite the differing suture material. This outcome highlights one of the inherent benefits of a locking stitch, which is to limit the amount of allowable displacement and may provide some load-sharing benefits that likely supersede the effects of varying suture materials in this scenario.

Several authors have reported biomechanical properties of graft fixation techniques using various stitch methods widely implemented in ACLR.^12^^,^^15^^,^^19^^,^^21^^,^^24^^,^^25^^,^^30^, ^31^, ^32^, ^33^, ^34^, ^35^, ^36^, ^37^ The commonly preferred suture techniques are the nonlocking whipstitch and the Krackow locking stitch, where the Krackow stitch has long been a gold standard.^18^^,^^19^^,^^21^^,^^38^ Clinical implications based on biomechanical characteristics are often unknown and difficult to infer; however, ultimate load to failure can be considered a critical biomechanical factor when choosing a graft as it represents the ability of a graft to withstand the anticipated load that will be experienced in daily activities postsurgery.^13^ Elongation has been associated with initial fixation to ensure graft tension is maintained until incorporation to native bone.^39^^,^^40^ The resistance to gap formation after repair is paramount for proper healing and favorable outcomes.^21^^,^^41^^,^^42^ Clinical threshold for failure has been cited as elongation greater than 3 mm in literature.^23^^,^^24^^,^^39^ Despite the differences detected between the groups, the peak-to-peak displacement for each group remained below the 3 mm clinical threshold.

Sakaguchi et al.^42^ evaluated the biomechanical properties of various stitch methods with a varying number of throws in porcine flexor tendon. One of their main outcomes of interest was that the Krackow group had superior biomechanical properties, resulting in less suture pullout, less elongation, and higher ultimate failure load, where most samples failed by rupture of the suture thread. Additionally, all specimens using a whipstitch method failed by suture pullout during cyclical testing. Steiner et al.^43^ also reported that the majority of their samples placed with a whipstitch method failed by suture-tendon stretching. Similar failure modes were observed in this study, highlighting one of the major limitations of the WS method thought to be caused by forces concentrated along the centerline of the tissue leading to suture cut-through (“cheese-wiring” effect).^15^^,^^19^ A recent study concluded that in small tendons, such as the ST, the LS is preferred for superior biomechanics and minimizing tissue pull-through, whereas in larger tendons, like the QT, either the WS or LS would provide sufficient biomechanical performance.^11^

An interesting observation in these study data was that the failure load for WS was higher than the LS for each group, but only group 1 was concluded to be statistically significant. This is contradictory to the outcomes published by Michel et al.,^40^ which reported Krackow stitch groups to have higher failure loads when compared to whipstitch groups in a human QT model. This discrepancy may be best explained by the difference in study design and methodology such as suture configuration, suture material, and loading protocol. They investigated various suture configurations using either 1 × No. 5 suture or 2 × No. 2 suture, whereas our study groups only utilized 1 × No. 2 suture. Moreover, the suture material, quantity, and size of suture may play a significant role in graft performance. Lastly, they implemented a different loading protocol, which began with a 10-N preload, followed by 500 cycles between 0 and 100 N, and then ramp to failure. Our protocol was 3 cycles of 25 to 100 N, followed by a 50-N hold for 1 minute, then 500 cycles between 50 and 200 N, and then ramp to failure. The inherent nature of the WS is that it concentrates force on the tissue (midline), while the LS spreads the load over the tissue and transfers it to the sutures. It is possible that in our study, the sutures failed sooner in the LS group than in the WS group, preventing the opportunity of achieving higher failure loads. Interestingly, the WS in group 2 and group 3 did have larger standard deviations, but this was minimized when the LS was performed. When looking across the manufacturers, the total elongation, stiffness, and ultimate load were found to be equivalent across all LS groups. The ultimate load for all groups (for both WS and LS) was above 300 N, a clinical threshold that an ACLR graft should sustain as the value represents the peak force exerted on the anterior cruciate ligament during the first quarter of the gait cycle.^44^, ^45^, ^46^ Furthermore, the stiffness of WS method group 2 was significantly larger than group 1, whereas stiffness of group 1 was equivalent to group 3.

The failure mode for WS method in group 1 occurred by suture breakage, minimizing tendon damage, whereas group 2 and group 3 experienced some failures related to suture pull-through. This is consistent with studies that demonstrate more abrasive properties in FiberWire,^15^^,^^47^^,^^48^ further supporting our findings related to group 2. There were 2 samples in group 3 that had experienced some suture pull-through before failing by suture breakage, and group 2 had 1 sample with complete suture pull-through. This failure mode is common across WS methods and occurs more frequently in less robust tendons such as rotator cuff repair.^7^^,^^47^^,^^49^^,^^50^ The LS method has been shown to minimize suture pull-through in varying quality of tendon grafts.^11^^,^^21^^,^^40^^,^^50^ This was also observed as the failure mode for the LS method was suture breakage across all groups.

A times savings of 56 seconds was noted for the WS in group 1 compared to group 2, where these seconds can compound over multiple surgeries in a day to save several minutes in the operating room; however, it is difficult to assess the practical significance of this finding. The participation of only 2 orthopaedic surgeons to prepare grafts makes it difficult to draw conclusions about stitching time. When times were compared across the 2 surgeons, there was a significant difference in their stitching times. Both surgeons were given the same amount of time to perform practice runs, but there were instances when the graft slipped from the preparation stand clamps, which may have contributed to differences in stitching time. Another factor to consider is the learning rate of the new stitching technique compared to the years of experience performing standard Krackow methods. While a larger sample size from multiple users would be required to properly quantify this learning curve, Park et al.^51^ published on the learning rate for the Krackow suture technique for the repair of Achilles tendon rupture. They concluded that when the cumulative volume of cases doubled, the required operative time could be decreased by up to 11%.

### Limitations

Several limitations of the present study are recognized. The average age of the cadaveric tissue used for this study was 73 years, which may not reflect the quality of soft tissue in a younger patient population receiving ACLR. As the quality and characteristics of soft tissue may decrease with age, this model can be considered a worst case. However, we found no significant differences between test groupings for age and the dimensions of tissue samples.

It should be noted that biomechanical evaluations cannot take into account factors such as tissue healing and thus only characterize properties time zero. Testing was conducted in a nonaqueous environment, but care was taken to maintain hydration with 0.9% saline throughout the duration of preparation and testing. This type of bench testing does not fully re-create the in vivo environment, but the protocol was developed to mimic early clinical loading of grafts. Pretensioning was performed to mimic graft preparation and implantation loading by surgeons, and preconditioning was also conducted to minimize viscoelastic creep. Loading was only performed in one direction. Grafts in vivo likely experience more complex loads in multiple directions, and prior research has demonstrated increased load to failure under physiological stress.^52^^,^^53^ The loading protocol in this study was developed based on previously published studies on similar tissue samples.^11^^,^^21^^,^^40^^,^^42^^,^^54^ Failure modes from the testing setup like knot or graft slippage from the testing machine are also possible, but these were not observed throughout testing in the current study.

Although the learning of surgical techniques cannot be applied consistently to all surgeons because of its subjective tendency, this learning curve may contribute to the time differences experienced.^51^

## Conclusions

Both LS and WS provide adequate mechanical properties in each of the 3 suture systems. Differences in performance do exist; however, each method shows equivalent total elongation and ultimate failure load for all 3 suture systems. LS may be preferred over WS due to lower mean elongation and failure displacement.

## Disclosures

The authors declare the following financial interests/personal relationships which may be considered as potential competing interests: This study was funded by Winter Innovations and supported by the National Science Foundation under Grant No. 2112103. M.A.D. received financial support by the Foundation for Orthopedic Research and Education and research grant Winter Innovations. A.B. has received speaking and lecture fees and travel reimbursement from Sanara MedTech and Zimvie. S.E.J. has received speaking and lecture fees and travel reimbursement Arthrex, CGG Medical, Vericel Corporation, GE Healthcare, Breg, and S-I Bone and has received consultation or advisory fees, funding or grants, and nonfinancial support from Arthrex and CGG Medical. All other authors (E.A.B., J.G.D.) declare that they have no known competing financial interests or personal relationships that could have appeared to influence the work reported in this paper.
